# Cross-talk between ER stress and mitochondrial pathway mediated adriamycin-induced testicular toxicity and DA-9401 modulate adriamycin-induced apoptosis in Sprague–Dawley rats

**DOI:** 10.1186/s12935-019-0805-2

**Published:** 2019-04-04

**Authors:** Keshab Kumar Karna, Bo Ram Choi, Jae Hyung You, Yu Seob Shin, Kiran Kumar Soni, Wan Shou Cui, Sung Won Lee, Chul Young Kim, Hye Kyung Kim, Jong Kwan Park

**Affiliations:** 1 Department of Urology, Institute for Medical Sciences, Chonbuk National University Medical School–Biomedical Research and Institute and Clinical Trial Center for Medical Devices, Chonbuk National University Hospital, Jeonju, 54907 Republic of Korea; 2 Department of Physiology & Biophysics, University of Mississippi Medical Center, Jackson, MS 39216 USA; 3 Andrology Center, Peking University First Hospital, Beijing, People’s Republic of China; 4 Department of Urology, Samsung Medical Center, Samsung Biomedical Research Institute, Sungkyunkwan University School of Medicine, Seoul, 06351 Republic of Korea; 5 College of Pharmacy, Hanyang University, Ansan, 426-791 Republic of Korea; 6 College of Pharmacy, Kyungsung University, Busan, 48434 Republic of Korea

**Keywords:** DA-9401, Adriamycin (ADR), Endoplasmic reticulum (ER) stress, Oxidative stress, Apoptosis, Steroidogenic acute regulatory protein (StAR), Cation channel of sperm (CatSper), Glycogen synthase kinase-3 (GSK-3), Blood-testis barrier (BTB)

## Abstract

**Background:**

DA-9401 was prepared as a mixture of Chinese medicinal herb extracts from roots of Morinda officinalis How (Rubiaceae), outer scales of *Allium cepa* L. (Liliceae) and seeds of Cuscuta chinensis Lamark (Convolvulaceae). The present study was designed to investigate the possible protective role of DA-9401 in adriamycin (ADR)-induced testicular toxicity associated with oxidative stress, endoplasmic reticulum (ER) stress, and apoptosis.

**Methods:**

Fifty healthy 8-week-old male Sprague–Dawley rats were equally divided into five groups. The first CTR group was treated with normal saline 2 ml/day by gavage. The second was treated with DA-100 (DA-9401 100 mg/kg/day). The third (ADR) group received ADR (2 mg/kg/once a week) intraperitoneally, while the combination of ADR and DA-9401 was given to the fourth ADR + DA-100 (100 mg/kg/day p.o) group and fifth ADR + DA-200 (200 mg/kg/day p.o) group. At the end of the 8-week treatment period, body weight, reproductive organ weights, fertility rate, pups per female were recorded, and serum were assayed for hormone concentrations. Tissues were subjected to semen analysis, histopathological changes, interleukin-6 (IL-6), tumor necrosis factor-α (TNF-α), oxidative stress markers and expression levels of endoplasmic reticulum (ER) stress markers, apoptosis markers, tight junction protein markers, steroidogenic acute regulatory protein (StAR), cation channel of sperm (CatSper) and glycogen synthase kinase-3 (GSK-3) by western blot.

**Results:**

DA-9401 administration to ADR-treated rats significantly decreased serum luteinizing hormone (LH) and follicle-stimulating hormone (FSH) levels, interleukin-6, TNF-α, MDA level, ROS/RNS level, ER stress response protein levels, tunnel positive cells, cleaved caspase-3, and Bax/Bcl2 ratio. Moreover, pretreatment with DA-9401 significantly increased body weight, reproductive organ weights, fertility rate, pups per female, Johnsen’s score, spermatogenic cell density, sperm count and sperm motility, serum testosterone concentration, testicular superoxide dismutase (SOD), catalase, glutathione peroxidase (GPx), tight junction protein markers, star protein level, CatSper, and GSK-3 level.

**Conclusions:**

ADR treatment can markedly impair testicular function and induce testicular cell death presumably by causing significant changes in oxidative stress, ER stress, and mitochondrial pathway. DA-9401 exerts beneficial effects against oxidative stress, ER stress, and mitochondria-mediated cell death pathway in testis tissue by up-regulating expression levels of tight junction protein markers, steroidogenic acute regulatory protein, GSK-3 alpha, and cation channels of sperm.

**Electronic supplementary material:**

The online version of this article (10.1186/s12935-019-0805-2) contains supplementary material, which is available to authorized users.

## Background

Adriamycin (ADR), an anthracycline-group antibiotic, is one of the most commonly used antineoplastic agents against a wide range of tumors such as hematological malignancy, ovarian cancer, breast cancer, and testicular cancer [1]. It inhibits DNA replication by intercalating into DNA strands and stabilizing topoisomerase II-DNA complex formation [2]. However, its current clinical use for long-term treatment is greatly limited by adverse side effects including delayed and progressive cardiomyopathy, nephrotoxicity, and testicular toxicity [3–5]. Treatment with ADR can reduce reproductive organ weight and lead to germ cell apoptosis, altered spermatogonial DNA, and decreased quality and quantity of sperm, thus significantly disturbing the fertility in adult rats [6, 7]. ADR-induced testicular toxicity differs from mechanisms responsible for its anti-tumor activity. ADR-induced testicular toxicity is caused by a combination of different pathophysiological events including oxidative stress, lipid peroxidation, mitochondrial dysfunction, increase tumor necrosis factor-α (TNF-α), and cellular apoptosis [3, 8]. Based on this concept, numerous studies in animals have tested various antioxidants and anti-apoptotic agents in an attempt to limit ADR-induced testicular damage [1, 2, 9–11].

DA-9401, a novel compound that acts as an antioxidant, is under development as treatment for male fertility. DA-9401 is a mixture of three medicinal herbs extracts [roots of *Morinda officinalis* How (Rubiaceae), seeds of C*uscuta chinensis* Lamark (Convolvulaceae), and outer scales of *Allium cepa* L. (Liliaceae)]. Monotropein, Deacetyl asperulosidic acid, Kaempferol 3-*O*-galactoside, quercetin, and quercetin 4′-*O*-glucoside are major compounds of DA-9401 [12]. DA-9401 exhibits various pharmacological activities such as antioxidant, anti-inflammatory, anti-nociceptive, androgenic, anti-stress, and anticancer effects [12]. DA-9401 is known to attenuate markers of oxidative stress, endoplasmic reticulum (ER) stress, and apoptosis level in Sprague–Dawley (SD) rats with subfertility induced by chronic administration of finasteride [12]. However, no data are available on effects of DA-9401 on ADR-induced infertility.

The current study aimed to evaluate pathophysiology, efficacy, and safety of DA-9401 and its possible correlation with ADR-induced testicular toxicity in different pathophysiological events, including oxidative stress, ER stress, and apoptosis markers in rat testis. In addition, western blot was performed for the testis to explore the role of tight junction protein markers, steroidogenic acute regulatory protein (StAR), glycogen synthase kinase-3 alpha (GSK-3α), and cation channels of sperm (CatSper) on DA-9401 mediated protection against ADR-induced testicular toxicity.

## Materials and methods

### Animals

The Animal Care and Ethics Committee of Chonbuk National University (cuh-IACUC-2017-10-2) approved all experiments. All efforts were made to minimize animal suffering. Fifty sexually mature male and female SD rats (weight: 210–240 g; age: 8 weeks) were obtained from KOATECH, Jeonwi-ro, Jinwei-myeon, Pyeongtaek-si, Gyeonggi-do, Korea. They received a standard rat chow diet with free access to water ad libitum. They were maintained in the animal facility at a constant room temperature of 20 ± 2 °C with relative humidity of 50 ± 10% and a 12-h light/dark cycle. Rats were acclimated to that environment for the first week. All rats were placed in plastic cages (47 × 18 × 40 cm) with four rats per cage.

### Chemicals and reagents

ADR was purchased from Tocris Bioscience (Tocris House, IO Center Moorend Farm Ave., Bristol, BS11 0QL, UK). All other chemicals were of analytical grade and purchased from standard commercial suppliers.

### Preparation of DA-9401

DA- 9401 was prepared as previously described [12].

### Experiential protocol

After 1-week of acclimatization, 8-week-old male SD rats weighing 220–240 g were randomly divided into five groups (10 rats per group): (1) control (CTR) group, (2) DA-9401 100 mg/kg/day p.o. group (DA 100), (3) ADR 2 mg/kg per week i.p. group (ADR), (4) ADR 2 mg/kg per week i.p. + DA-9401 100 mg/kg/day p.o. (ADR + DA 100), and (5) ADR 2 mg/kg per week i.p. + DA-9401 200 mg/kg p.o. group (ADR + DA 200). DA-9401 was dissolved in two different containers with sterile normal saline and administrated orally by gavage with a Zonde needle **(**JD-S-124, Jeungdo, Seoul, Korea) at a single dose of 100 or 200 mg/kg/day. The CTR group received normal saline (vehicle) for 56 days. ADR was dissolved in distilled water and received 2 mg/kg intraperitoneally once a week for 56 days. This dose is well documented to be able to induce testicular toxicity in rats [4]. Fifty female rats were used to determine fertility parameters after natural mating. Each mating pair was kept in single case after 6 weeks of medication. After 2 weeks, female rats were separated from male rats and kept in a separate singled case. Male rats were proven fertility by producing offspring. All male rats were anesthetized 48 h after the last treatment. Rats were anaesthetized with mixture of ketamine (100 mg/ml) and 2% rumpin (20 mg/ml) at a dose of 170–230 μl/100 gm body weight [13]. Blood samples were collected from rats’ vena cava. Testis tissues were collected and used for the following analysis.

### Reactive oxygen species (ROS)/reactive nitrogen species (RNS) and malondialdehyde (MDA) level

ROS/RNS assay was determined using a fluorescence kit (STA-347, OxiSelect^TM^ in vitro ROS/RNS assay kit, Cell Biolabs, Inc., San Diego, CA, USA) at excitation and emission wavelengths of 480 and 530 nm, respectively, with a SpectraMax Gemini XS Fluorimeter. To assess lipid peroxidation in SD rat testes, MDA levels in testis tissue homogenates were measured using a commercially available kit (NWLSSTM Malondialdehyde Assay kit; Northwest Life Science Specialties LLC., Vancouver, WA, USA) following the manufacturer’s instructions. MDA forms a pink complex in aerobic conditions after incubation with thiobarbituric acid (TBA) at 60 °C. Absorbance of the colored complex was measured by kinetic spectrophotometric analysis at 532 nm using a Spectra Max 180 (Molecular Devices, Sunnyvale, CA, USA). MDA concentration in the sample was analyzed by comparing the measured absorbance value to an MDA standard curve [14]. MDA concentrations were normalized to total protein content [15].

### Sperm motility and sperm count in the vas deferens and epididymis

The distal cauda of the epididymis and the entire length of the vas deferens were removed and placed in separate microcentrifuge tubes, minced, and suspended in pre-warm normal saline at 37 °C for 5 min. Sperm motility was evaluated by observing a sperm suspension within 3–5 min after being placed on a pre-warmed counting chamber (SEFI-Medical Instruments, Haifa, Israel). The number of motile spermatozoa within 10 squares of the grid were counted under a light microscope and mean sperm count was recorded. The percentage of motile spermatozoa was determined with the following formula: (mean number of motile spermatozoa/total number of spermatozoa) × 100%.

### Spermatogenic cell density and Johnsen’s score

Testes tissues were immediately fixed in Bouin’s solution for 48 h and dehydrated through a graded ethanol series. These tissue samples were embedded in paraffin, sectioned (5 μm in thickness), deparaffinized, rehydrated, and stained with hematoxylin and eosin (H&E). Testis tissue was evaluated using standard light microscopy. Ten seminiferous tubules (ST) were randomly examined per section. Their diameters and germinal cell layer thicknesses (from the basal membrane towards the lumen of the tubule) were measured using an image analysis program (i-Solution; IMT i-solution Inc., Vancouver, BC, Canada). Spermatogenic cell density was determined by measuring the thickness of the germinal cell layer and the diameter of the seminiferous tubules. The seminiferous tubules of H&E-stained sections at X400 were graded by Johnsen’s score as previously described [13]. Damaged tubules at edges of the section were excluded. A minimum of 20 seminiferous tubules from these slides were assessed according to the presence of spermatogenic cells and assigned a score from 1 to 10.

### Terminal deoxynucleotidyl transferase-mediated (dUTP) nick-end labeling (TUNEL) staining

Small pieces of testis tissue from each group were fixed in Bouin’s solution in phosphate-buffered saline (PBS) and then processed via dehydration in a graded ethanol series, embedded, and sectioned at 5 µm in thickness on the serial coronal plane. Apoptotic activity within the seminiferous tubules was determined using TUNEL assays (Dead End™ Colorimetric TUNEL System for qualitative study; Promega, Madison, WI, USA). All procedures were carried out according to the manufacturer’s instruction. Two slides from each animal were used for quantitative study. In cross section, 100 seminiferous tubules from each group were counted for the number of apoptotic cells under a fluorescence microscope (20× objective). Positive nuclei stained dark-brown were visualized under a light microscope.

### Determination of hormonal assay

Levels of sex hormones including serum testosterone, luteinizing hormone (LH), and follicle-stimulating hormone (FSH) were measured by enzyme-linked immunosorbent assay (ELISA) using commercial kits (55-TESMS-E01, Mouse/rat testosterone kit, ALOCO, 26-G Keewaydin Drive, Salem, NH, USA; E-EL-R0026, rat LH Elisa kit; E-EL-R0391, rat FSH Elisa kit; Elabscience, Houston, Texas, USA) following manufacturers’ instructions.

### Determining concentrations of superoxide dismutase (SOD), glutathione peroxidase (GPx), and catalase

Testis tissues (100 mg) were rinsed with 1X PBS (pH 7.4) to remove any red blood cells and clots. Concentrations of SOD, GPx, and catalase in whole tissue supernatant were measured using commercial kits (item no. 706002, superoxide dismutase kit; item no. 703102, glutathione peroxidase kit; item no. 707002, catalase assay kit, Cayman Chemical, Ann Arbor, MI, USA) following the manufacturer’s instructions. Values are expressed as per milligram protein.

### Cytokines measurements

Testis tissues (100 mg) were rinsed with 1X PBS (pH 7.4) to remove any red blood cells and clots. Tissues in 1 ml of 1X PBS were homogenized with a homogenizer on ice and stored at − 20 °C overnight. Two freeze–thaw cycles were then performed and the homogenate was centrifuged at 10,000×*g* for 15 min at 4 °C. The supernatant was used for assays. Concentrations of interleukin-6 (IL-6) and TNF-α were measured by enzymatic method using commercial kits (BMS625 IL-6 rat Elisa kit, BMS 622 rat TNF-α kit, Thermo Fisher Scientific, Waltham, MA, USA) following the manufacturer’s instructions. Values are expressed as per milligram protein.

### Western blotting

Testis tissues were washed twice with cold PBS and homogenized using a cordless motor pellet pestles in extraction buffer (50 mM Tris–HCl pH 7.4, 150 mM NaCl, 1 mM EDTA, 1% Triton X-100 and 0.1% SDS) supplemented with protease inhibitor for 30 min on ice. Tissue lysates were centrifuged at 13,000×*g* for 30 min at 4 °C. The supernatant was collected and stored at − 80 °C. Protein concentration was determined by Bradford protein assay. Levels of ER stress marker [glucose-regulated protein-78 (GRP-78), phosphorylated inositol-requiring transmembrane kinase/endoribonuclease 1α (p-IRE1α), phosphorylated c-Jun-N-terminal kinase (p-JNK)], apoptosis markers [pro-caspase-3, cleaved caspase 3, BCL 2 associated X protein (Bax), B cell lymphoma 2 (Bcl-2)], steroidogenic acute regulatory protein (StAR), cation channels of sperm (CatSper), glycogen synthase kinase-3 (GSK-3), occludin, claudin 11, and zonula occludens-1 (ZO-1) were measured in testis tissue. A total of 30–60 μg of extracted protein from each sample was loaded per lane, subjected to 8–12% SDS–polyacrylamide gel electrophoresis, and electro blotted onto PVDF membranes with a trans-blot^®^ SD semi-dry electrophoretic transfer cell (Bio-Rad, Hercules, CA, USA). For higher molecular weight proteins, overnight wet transfer was performed at 20 V. After protein transfer, the membrane was blocked with 5% bovine serum albumin (BSA) for an hour at room temperature and incubated with the following primary antibodies overnight at 4 °C: phosphorylated antibodies p-IRE1α (Abcam Cambridge, MA USA) and p-JNK (Santa Cruz Biotechnology, Dallas, TX, USA), non-phosphorylated antibodies GRP-78, pro-caspase-3, cleaved caspase 3, Bax, Bcl-2, StAR (Cell Signaling Technology, Beverly, MA, USA), occludin (Abcam Cambridge, MA USA), claudin 11, ZO-1 (Santa Cruz Biotechnology, Dallas, TX, USA), and GSK-3 (Thermo Fisher Scientific, Waltham, MA, USA) in the presence of 5% non-fat milk. The membrane was washed with Tris-buffered saline containing 0.05% Tween 20 (TBST, pH 7.2) three times prior to incubation with 1:5000 diluted secondary antibody [anti-mouse, anti-rabbit (Cell Signaling Technology, Beverly, MA, USA), or anti-rat IgG (Santa Cruz Biotechnology, Dallas, TX, USA)] at room temperature for 1 h. The membrane was washed three times with TBST. Antigen–antibody complexes were then visualized with an ECL system (Vilber Lourmat, France).

### Statistical analyses

All data are expressed as mean ± standard error of the mean (SEM). A *P*-value < 0.05 was considered statistically significant by one-way analysis of variance (ANOVA) followed by Tukey’s post hoc test. GraphPad PRISM (Version 5, GraphPad Software, San Diego, CA, USA) was used for the graph analysis. Statistical calculations were performed using SPSS version 22 (IBM, Armonk, NY, USA).

## Results

### Body weight, organ weights, serum hormone concentrations, sperm count, sperm motility, and fertility parameters

Effects of DA-9401 on body weight, organ weights, serum concentration of testosterone, LH and FSH levels, sperm count, sperm motility in vas deference and epididymis, and fertility parameters are summarized in Table 1. Body weight and reproductive organ weight, serum testosterone, sperm count, sperm motility in both vas deference and epididymis, fertility rate and pups per female were significantly decreased in the group with ADR administration compared to those in the control (all *P *< 0.05). Treatment with DA-9401 to ADR-administered group significantly increased body weight, organ weight, serum testosterone, sperm count and motility in vas deference and epididymis, fertility rate, and pups per female compared to ADR group (*P *<0.05). Serum LH and FSH levels were significantly increased (*P *<0.05) in the ADR group compared to those in the CTR group. However, a significant decrease in serum LH level was noted in ADR + DA 200 group. A trend toward a decrease in serum LH level in ADR + DA 100 group was noted, although the decrease was not statistically significant. Serum FSH level was significantly decreased (*P *<0.05) in ADR + DA 100 and ADR + DA 200 groups compared to that in the CTR group. There were no significant difference in body weight, serum hormone concentrations, sperm count, sperm motility and fertility parameters between ADR + DA 100 and ADR + DA 200 groups. Similarly no difference in organ weight except prostate weight is significantly decreased with compare to ADR + DA 100 group.

**Table 1:** Effect of DA-9401 on body, reproductive organ weight, serum hormone concentration, sperm count, motility, and fertility parameters in adriamycin treated male SD rats

Parameter	CTR	DA 100	ADR	ADR + DA 100	ADR + DA 200
Body and organ weight					
Body weight (beginning; gm)	229.00 ± 3.97	225.10 ± 3.41	235.91 ± 3.66	239.22 ± 5.37	232.00 ± 2.10
Body weight (sacrifice; gm)	413.40 ± 9.12	409.20 ± 9.15	320.80 ± 7.94*^#^	369.88 ± 13.61*^#+^	341.50 ± 7.98*
Testis weight (gm)	2.03 ± 0.05	2.06 ± 0.02	0.81 ± 0.05*^#^	0.91 ± 0.03*^#^	1.14 ± 0.12*^+^
Epididymis weight (gm)	0.72 ± 0.14	0.71 ± 0.01	0.48 ± 0.02*^#^	0.59 ± 0.02*^#+^	0.56 ± 0.03*^+^
Seminal vesicles weight (gm)	1.34 ± 0.04	1.45 ± 0.05	0.66 ± 0.06*^#^	1.03 ± 0.08*^#+^	0.94 ± 0.07*^+^
Prostate weight (gm)	0.97 ± 0.05	1.16 ± 0.07	0.67 ± 0.05*^#^	0.90 ± 0.04^#+^	0.66 ± 0.04*^$^
Penis weight (gm)	0.31 ± 0.01	0.31 ± 0.01	0.26 ± 0.01*^#^	0.30 ± 0.01^+^	0.28 ± 0.01
Serum hormone levels					
Serum testosterone (ng/ml)	5.36 ± 0.96	5.53 ± 0.58	0.42 ± 0.07*^#^	1.62 ± 0.13*^#^	2.75 ± 0.51*^+^
Serum LH (mIU/ml)	39.10 ± 4.07	46.17 ± 2.41	101.26 ± 8.67*^#^	84.33 ± 11.60*^#^	88.27 ± 10.62*
Serum FSH (ng/ml)	4.07 ± 0.58	4.57 ± 0.88	14.30 ± 1.68*^#^	4.41 ± 1.85^+^	4.41 ± 0.23^+^
Sperm count and motility					
Sperm count (10^6^/ml)					
Vas deference	29.14 ± 0.99	26.64 ± 1.82	17.35 ± 0.89*^#^	22.42 ± 0.79*^#+^	22.28 ± 0.28*^+^
Epididymis	37.78 ± 1.28	34.78 ± 0.79	10.42 ± 2.01*^#^	16.85 ± 0.96*^#+^	18.28 ± 1.64*^+^
Sperm motility (%)					
Vas deference	65.72 ± 3.06	60.60 ± 4.67	31.50 ± 2.12*^#^	48.13 ± 2.72*^+^	39.99 ± 1.85*
Epididymis	36.78 ± 1.45	38.92 ± 2.19	15.33 ± 1.71*^#^	26.55 ± 1.65*^#+^	22.57 ± 1.50*^+^
Fertility parameters					
Fertility rate (%)	100	100	50*^#^	80	100^+^
Pups per female	12.80 ± 0.78	11.90 ± 0.56	5.60 ± 1.96*^#^	9.3 ± 1.78	10.50 ± 0.85^+^

Results are expressed as mean ± SEM

CTR, control; DA 100, DA-9401 100 mg/kg/day p.o.; ADR, adriamycin 2 mg/kg i.p. per week; ADR + DA 100, adriamycin 2 mg/kg i.p. per week + DA-9401 100 mg/kg/day p.o.; ADR + DA 200, adriamycin 2 mg/kg i.p. per week + DA-9401 200 mg/kg/day p.o. LH, luteinizing hormone; FSH, follicle stimulating hormone; p.o., per oral; i.p., intraperitoneally

*P < 0.05 vs. CTR group, ^#^*P* < 0.05 vs. DA 100 group, ^+^*P* < 0.05 vs. ADR group, ^$^*P* < 0.05 vs. ADR + DA 100 group (one-way ANOVA followed by the Tukey post hoc test; n = 10 for each group)

### Levels of lipid peroxidation, antioxidant enzymes, and cytokines in testis tissues

Levels of MDA, ROS/RNS, SOD, GPx, catalase, IL-6, and TNF-α in testicular tissue are summarized in Table 2. Levels of MDA, ROS/RNS, IL-6, and TNF-α were significantly (*P *< 0.05) increased in the ADR administration group compared to those in the CTR group. Treatment with DA 100 and DA 200 significantly (*P *<0.05) decreased these parameters compared to the ADR group. ADR induced significant decrease in SOD, GPx, and catalase levels compared to the CTR group. These levels were significantly (*P *<0.05) increased after combine treatment of DA 100 and DA 200 with ADR. No significant results were observed between ADR + DA 100 and ADR + DA 200 group.

**Table 2:** Effect of DA-9401 on biomarkers of oxidative stress and inflammatory in adriamycin treated male SD rats

Parameter	CTR	DA 100	ADR	ADR + DA 100	ADR + DA 200
MDA (μmol/mg protein)	6.83 ± 1.16	8.45 ± 0.70	14.75 ± 2.87*	7.99 ± 1.25^+^	3.40 ± 0.35^+^
ROS/RNS (nanomole DCF/mg protein)	458.55 ± 17.71	397.75 ± 67.78	753.61 ± 30.71*^#^	300.91 ± 26.15^+^	296.65 ± 59.45^+^
SOD level (units/mg protein)	6.29 ± 0.56	6.26 ± 0.32	2.76 ± 0.10*^#^	5.99 ± 0.46^+^	4.80 ± 0.13^+^
GPx (nanomoles/min/mg protein)	44.29 ± 3.72	51.47 ± 3.42	18.20 ± 1.56*^#^	40.47 ± 5.08^+^	50.09 ± 3.88^+^
Catalase (nanomoles/min/mg protein)	116.88 ± 6.51	122.83 ± 9.01	65.01 ± 2.48*^#^	132.21 ± 18.02^+^	112.06 ± 6.90^+^
IL-6 (pg/mg protein)	1264.93 ± 32.16	1336.43 ± 129.61	3685.30 ± 497.63*^#^	1569.39 ± 370.72^+^	1341.60 ± 109.25^+^
TNF-α (pg/mg protein)	1174.73 ± 55.29	1197.43 ± 152.88	3182.75 ± 232.87*^#^	1739.86 ± 182.09^+^	1590.79 ± 107.5^+^

Results are expressed as mean ± SEM

CTR, control; DA 100, DA-9401 100 mg/kg/day p.o.; ADR, adriamycin 2 mg/kg i.p. per week; ADR + DA 100, adriamycin 2 mg/kg i.p. per week + DA-9401 100 mg/kg/day p.o.; ADR + DA 200, adriamycin 2 mg/kg i.p. per week + DA-9401 200 mg/kg/day p.o. MDA, malondialdehyde; ROS/RNS, reactive oxygen species/reactive nitrogen species; SOD, superoxide dismutase; GPx, glutathione peroxidase; IL-6, interleukin-6; TNF-α, tumor necrosis factor- α; p.o., per oral; i.p., intraperitoneally

* *P* < 0.05 vs. CTR group, ^#^*P* < 0.05 vs. DA 100 group, ^+^*P* < 0.05 vs. ADR group (one-way ANOVA followed by the Tukey post hoc test; n = 10 for each group)

### Testicular histology and maturity of the germinal epithelium

**Fig. 1:**
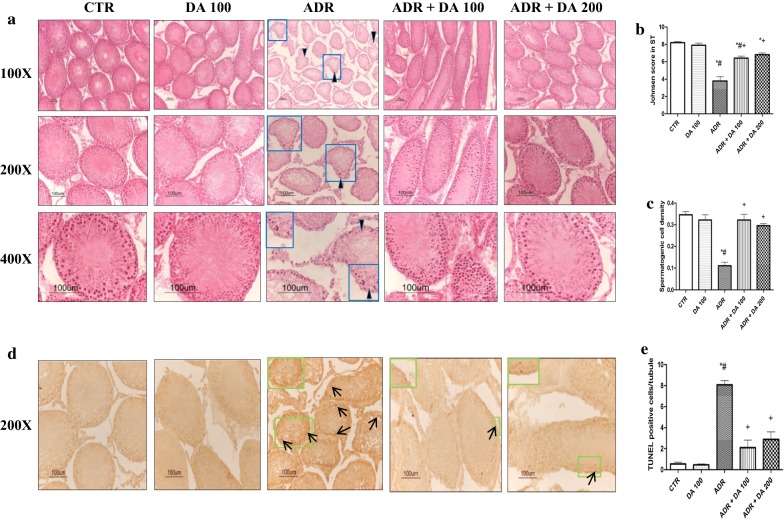
Effect of DA-9401 on microscopic observations and histological analysis with hematoxylin and eosin (H&E) staining and terminal deoxynucleotidyl transferase-mediated dUTP nick end labeling (TUNEL) staining of the testis of adriamycin (ADR)-treated male SD rats. **a** Rat testis cross sections stained with H&E showing loss of spermatogenic cells and seminiferous tubule vacuole with disorganization in the ADR group (arrowhead; **a**). **b** Johnsen score in seminiferous tubules. **c** Spermatogenic cell density in seminiferous tubules. **d** Cross section of TUNEL stained testis to show TUNEL positive cells (arrows; **d**). **e** Quantification of TUNEL-positive cells analyzed as total positive cells/seminiferous tubule. Results are expressed as mean ± SEM. CTR, Control; DA 100, DA-9401 100 mg/kg/day p.o.; ADR, Adriamycin 2 mg/kg i.p. per week; ADR + DA 100, Adriamycin 2 mg/kg i.p. per week + DA-9401 100 mg/kg/day p.o.; ADR + DA 200, Adriamycin 2 mg/kg i.p. per week + DA-9401 200 mg/kg/day p.o.; p.o, Per oral; i.p., Intraperitoneally. *P < 0.05 vs. CTR group, ^#^*P *< 0.05 vs. DA 100 group, ^+^*P *< 0.05 vs. ADR group (one-way ANOVA followed by Tukey’s post hoc test; n = 10 for each group)

Histological analysis of H&E stained testis section showed no remarkable histological findings in the CTR group. However, significant degenerative changes in germinal epithelium of the seminiferous tubules associated with atrophy, immature germinal cell, vacuolation (Fig. 1a), apoptosis (Fig. 1d), and high number of TUNEL positive cells (Fig. 1e) were detected in the ADR group (*P *<0.05). Johnsen’s score and spermatogenic cell density were significantly (*P *<0.05) decreased in the ADR group compared to those in the CTR group (Fig. 1b, c). However, such ADR-induced alterations in testicular histology were not found in DA-100 only group. They were effectively reverted in DA-9401 pretreated groups (ADR + DA 100 group and ADR + DA 200 group) (Fig. 1a–e). No significant results were observed between ADR + DA 100 and ADR + DA 200 group.

### Western blot studies of protein expression in testis tissue

**Fig. 2:**
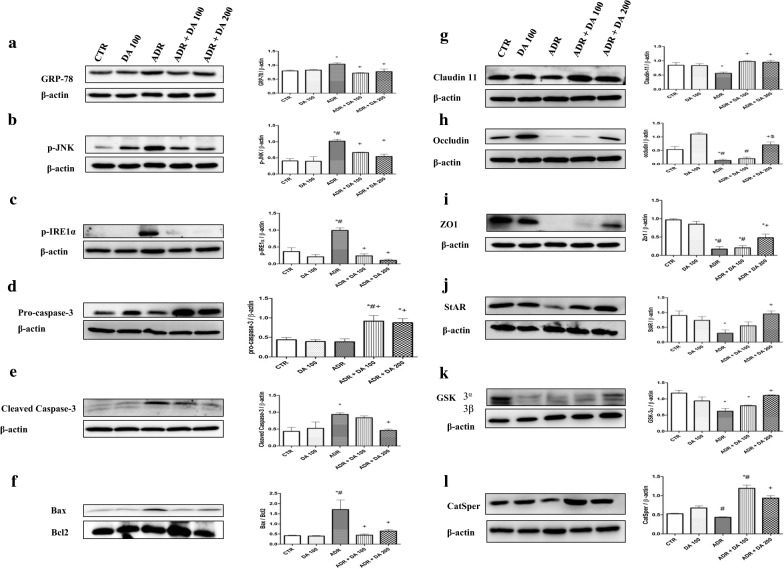
Effect of DA-9401 on testicular protein levels in adriamycin (ADR) treated male SD rats determined by western blot analysis. **a** GRP-78, **b** p-JNK, **c** p-IRE, **d** Pro-caspase-3, **e** Cleaved caspase-3, **f** Bax/Bcl2 ratio, **g** Claudin 11, **h** Occludin, **i** ZO1, **j** StAR, **k** GSK3, **l** CatSper. Results are expressed as mean ± SEM. CTR, Control; DA 100, DA-9401 100 mg/kg/day p.o.; ADR, Adriamycin 2 mg/kg i.p. per week; ADR + DA 100, Adriamycin 2 mg/kg i.p. per week + DA-9401 100 mg/kg/day p.o.; ADR + DA 200, Adriamycin 2 mg/kg i.p. per week + DA-9401 200 mg/kg/day p.o.; GRP-78, Glucose regulated protein-78; p-JNK, Phosphorylated c-jun-N-terminal kinase; p-IRE1α, Phosphorylated Inositol-Requiring Transmembrane Kinase/Endoribonuclease 1α; Bax, BCL 2 associated X protein; Bcl-2, B-cell lymphoma 2; ZO1, Zonula occludens-1; StAR, Steroidogenic acute regulatory protein; GSK-3α, Glycogen synthase kinase 3α; CatSper, Cation channels of sperm; p.o., Per oral; i.p., Intraperitoneally. *P < 0.05 vs. CTR group,^#^*P *< 0.05 vs. DA 100 group, ^+^*P *< 0.05 vs. ADR group, ^$^*P* < 0.05 vs. ADR + DA 100 group (one-way ANOVA followed by Tukey’s post hoc test; n = 10 for each group)

To assess the effect of DA-9401 on ADR induced testicular toxicity by ER stress-mediated cell death pathway; levels of GRP-78, p-IRE1α, and p-JNK were evaluated in the testis of SD rats (Fig. 2a–c). Administration of ADR significantly (*P *<0.05) increased levels of GRP-78, p-IRE1α, and p-JNK compared to the CTR group whereas levels of GRP-78, p-IRE1α, and p-JNK levels in ADR + DA 100 and AD + DA 200 groups were significantly (*P *<0.05) lower than those in the ADR group. Levels of pro-caspase-3 were not significantly different between the ADR group and the CTR group, although they were significantly (*P *<0.05) increased in ADR + DA 100 and ADR + DA 200 groups than those in the ADR group (Fig. 2d). Levels of cleaved caspase-3 were significantly (*P *<0.05) increased in the ADR group, but significantly (*P *<0.05) decreased in the ADR + DA-200 group compared to those in the CTR group (Fig. 2e). A trend toward a decrease in cleaved caspase-3 expression level was noted in ADR + DA 100 group compared to the ADR group, although the decrease was not statistically significant. Mitochondrial cell death pathway was analyzed by examining the ratio of Bax and Bcl2 expression (Fig. 2f). A ratio of Bax and Bcl2 expression was significantly (*P *<0.05) increased in the testis of the ADR group compared to that of the CTR group. However, it was significantly (*P *<0.05) decreased in DA-9401 treated groups (ADR + DA 100 group and ADR + DA 200 group) to normal levels of Bax/Bcl2 expression ratio.

Degradation of tight and adherent junction protein is one of critical events associated with leaking of Sertoli cell barrier (SCB) which facilitates passage of cytotoxic agents into the seminiferous tubules. Western blot results for key tight junction proteins such as claudin-1, occludin, and Zo-1 are shown in Fig. 2g–i. Claudin-1, occludin, and ZO-1 levels were significantly (*P *<0.05) decreased in the ADR group compared to those in the CTR group, but not significantly changed in the DA-100 group. In contrast, DA-9401 significantly (*P *<0.05) upregulated levels of claudin-1, occludin, and ZO-1 in a dose-dependent manner, especially in the ADR + DA 200 group compared to those in the ADR group. Furthermore, a decline in steroidogenesis was evident, showing significant (*P *<0.05) reduction in expression level of steroidogenic marker such as StAR in ADR group compared to the CTR group (Fig. 2j). In contrast, compared to the ADR group, DA-9401 up-regulated (*P *<0.05) StAR expression level in a dose-dependent manner, especially in ADR + DA 200 group. GSK-3α and CatSper activity are known to correlate with motility of sperm. Protein expression level of GSK-3α was significantly (*P *<0.05) decreased in the ADR group compared to that in the CTR group (Fig. 2k). However, it was significantly (*P *<0.05) increased in the ADR + DA 200 group compared to that in the ADR group. A trend toward an increase in GSK-3α expression level was noted in the ADR + DA 100 group compared to that in the ADR group, although the difference was not statistically significant. The expression level of CatSper was downregulated in the ADR group, but significantly (*P *<0.05) up-regulated after treatment with DA-9401 (in both ADR + DA 100 group and ADR + DA 200 group, Fig. 2l). No significant results were observed between ADR + DA 100 and ADR + DA 200 group except the expression level of occludin protein.

## Discussion

ADR based anticancer therapy can damage to heart, kidney, liver, brain, reproductive organs, and so forth [16]. Many studies have reported that ADR could trigger impairment in testicular function in male rats [3, 8]. ADR can significantly increase oxidative stress and lipid peroxidation of testicular tissue, cause alteration in testicular histology, decrease sperm parameters, and increase apoptosis in rats [1, 2, 17]. Furthermore, ADR can significantly alter gonadotropin (LH, FSH) and testosterone levels known to be prime regulators of germ cell development [3, 4, 18, 19]. It has been reported that DA-9401 is an effective antioxidant that can scavenge free radicals, reduce lipid peroxidation, and ameliorate ER stress-mediated testicular apoptosis in rats [12]. Therefore, we chose DA-9401 as a protective agent against ADR-induced testicular toxicity in the present study.

Results of the present study indicated that SD rats treated with ADR showed significant decreases in body and reproductive organ weight, consistent with previous findings reporting that treatment with ADR could reduce body weight and reproductive organ weight in rats [7, 17]. However, treatment with DA-9401 improved body weight and reproductive organ weight. We observed that sperm count, sperm motility, and serum concentration of testosterone were significantly decreased in the ADR group, similar to results of previous studies in rats [20, 21]. Testosterone plays an essential role in male fertility and spermatogenesis as well as maintenance of structural morphology and normal physiology of seminiferous tubules [22, 23]. These studies suggest that ADR has direct inhibitory effect on androgen biosynthesis in Leydig cells. The significant reduction in sperm count and motility might be due to increase in toxin ROS which is a crucial factor in sperm DNA fragmentation and sperm motility [24, 25]. This study also demonstrates that DA-9401 can improve sperm count, sperm motility, and serum testosterone level. In this study, serum levels of LH and FSH were increased in the ADR group. Gonadal toxicity involving spermatogenic damage is normally associated with alteration in serum FSH and LH levels. However, a change in pituitary-Leydig cell axis in human is not clearly understood yet [19, 26]. In the present study, treatment with DA-9401 decreased serum levels of LH and FSH. Fertility rate, and pups per female were downregulated in ADR groups. However, DA-9401 optimized these parameters. In addition, histological examination revealed that ADR decreased germ cell proliferation, spermatogenic cell density, and Johnsen’s score in seminiferous tubules while these effects were reversed by DA-9401 pre-treatment.

Several studies have reported that testicular toxicity caused by ADR is due to generation of oxidative stress, increased inflammatory cytokines, and apoptosis while many anti-oxidant or anti-inflammatory agents could ameliorate such toxicity effects in rats [8, 21, 23, 27]. Oxidative stress is characterized by increased generation of MDA and elevated expression of antioxidants. Increased ROS impairs spermatogenesis by peroxidation of membranous lipids and fragmentation of nucleic acids [23]. Oxidative stress in the ADR treated group was evident, showing increased level of testicular MDA and ROS levels with suppressed activity of antioxidant enzymes such as SOD, catalase, and GPx. However, treatment with DA-9401 restored all these parameters in ADR-exposed rats. The protection rendered by DA-9401 was due to its free radical scavenging ability and antioxidant activity [12]. ADR-induced toxicity has a strong association with oxidative stress and inflammatory response, including upregulation of cytokines [8]. Monotropein from roots of *Morinda officinalis* is a major compound of DA-9401. It has been previously reported to possess anti-inflammatory activities [28]. The present study confirmed that DA-9401 possessed anti-inflammatory properties because treatment with DA-9401 in ADR-exposed rat decreased levels of IL-6 and TNF-α compared to those in the ADR group.

Increase ROS production has a bidirectional relationship with oxidative stress and ER stress, leading to accumulation of unfolded protein in the ER [29]. Interruption of ER function by oxidative stress, iron imbalance, Ca^2+^ leakage, protein overload, and hypoxia can cause ER stress and lead to accumulation of unfolded or misfolded proteins and apoptosis [30]. Prolonged ER stress can lead to apoptosis which is mainly mediated by PERK and IRE1 signaling pathways [31]. For the first time, the present study found that long-term treatment with ADR could activate unfolded protein response (UPR) signaling in testis by IRE1 mediated ER stress pathway. IRE1α is known to stimulate activation of apoptotic signaling kinase-1 (ASK1) that is required for ROS and ER stress-induced JNK activation and apoptosis [32, 33]. First, GRP-78, an important ER molecule chaperone, was significantly increased in the testis of ADR-treated rat. Second, p-IRE 1α, a downstream target of the IRE-1α signaling pathway, was significantly increased, indicating that IRE1 pathway was activated by ADR. Finally, JNK, a downstream target of an IRE1 pathway, was analyzed. Levels of p-JNK in ADR-treated rats were upregulated. Increase in p-JNK in the ADR group was similar to previous findings [23]. Thus, treatment with DA-9401 inhibited ER stress by downregulating IRE 1-JNK signaling pathway.

ADR is a well-known pro-apoptotic agent in male germ cells of rat model. It is predominantly mediated by the mitochondrial cell death pathway [34]. Apoptosis in ADR treated group was evident in the present study, showing increased levels of TUNEL positive cells and cleaved caspase-3 protein expression level. A previous study has shown that JNK could promote translocation of Bax from cytosol to the mitochondria through direct phosphorylation of Bax [35] or phosphorylation of 14-3-3, a cytoplasmic anchor of Bax [36]. Bax plays an important role in consequent release of mitochondrial cytochrome c into the cytosol and subsequent apoptosis [34]. In our study, the mitochondrial cell death pathway was examined by a ratio of Bax to Bcl2 expression level which was upregulated in the ADR group. The present study showed that apoptosis was reversed by DA-9401 pre-treatment. A previous study has shown that cadmium can induce testicular germ cell apoptosis by ER stress signaling and mitochondrial pathway [37]. Moreover, the present study suggests that a crosstalk between ER stress and mitochondrial cell death pathway can mediate ADR-induced testicular germ cell apoptosis.

Another main objective of the present study was to explore if the blood-testis barrier (BTB) might play a key role in testicular toxicity mechanism of ADR. BTB injury can cause germ cell loss and reduce sperm count and male infertility [38]. BTB is formed largely by the tight junction between Sertoli cells that are crucial for spermatogenesis. Damage in Sertoli cells can lead to germ cell apoptosis [39]. A previous study has reported that BTB injury by ADR is mediated by the generation of free radicals and lipid peroxidation [6]. However, ADR-induced damage of BTB during puberty remains obscure. The current study demonstrated that the integrity of tight junction in rat testis of ADR-treated group was substantially damaged. Tight junction proteins such as cloudin-11, occludin, and ZO1 play a critical role in maintaining BTB integrity [40]. Expression levels of BTB junction proteins claudin-11, occludin, and ZO1 were downregulated in the ADR group. However, DA-9401 administered concomitantly with ADR reversed these parameters.

Finally, decrease in testosterone and sperm motility by ADR was further confirmed by analyzing protein expression levels of StAR, GSK-3α, and CatSper. StAR is a prime regulatory protein for testosterone biosynthesis in the testis. StAR plays a key role in transportation of cholesterol from its intracellular location into mitochondrial inner membrane [8]. CatSper is a key ion transport protein of sperm. It is vital to cAMP-mediated calcium influx in sperm, sperm motility, and fertilization. The present study showed downregulation of StAR and CatSper in the ADR-treated group, similar to previous finding in cisplatin-treated rats [30]. Signaling kinase GSK-3α, a predominant isoform of GSK-3, is present in the testis [41]. The present study showed that both isoforms of GSK-3 (GSK-3α and GSK-3β) were present in the testis, with GSK-3α being the predominant isoform. Changes in sperm glycogen synthase kinase-3α serine phosphorylation by upstream signaling enzymes cAkt and PI3-kinase play a key role in sperm motility [42]. In the present study, GSK-3α expression level was downregulated in the ADR-treated group. However, DA-9401 administered concomitantly with ADR increased expression levels of StAR, CatSper, and GSK-3α.

## Conclusion

In summary, as shown in Additional file 1: Fig. S1, the present study indicated that DA-9401 showed protective effects against ADR-induced testicular toxicity in SD rats, exhibiting free radical scavenging effect. DA-9401 improved sperm count and sperm motility. It also exerted androgenic and anti-inflammatory activities. These results support that cross-talk between ER stress and mitochondrial cell death pathway can mediate ADR-induced testicular germ cell apoptosis. Furthermore, the present study provided important new insights into the role of oxidative stress and BTB in aggravation of testicular damage caused by ADR. Thus, DA-9401 might represent a promising therapeutic modality to ameliorate ADR-induced testicular toxicity.

## Additional file

**Additional file 1: Fig. S1.** Schematic diagram showing adriamycin (ADR)-induced testicular toxicity and its prevention by DA-9401 via suppression of oxidative stress, inflammation, endoplasmic reticulum (ER) stress, blood testis barrier (BTB), and apoptosis in the testis tissue. ER: Endoplasmic reticulum; ROS/RNS: Reactive oxygen species/reactive nitrogen species; MDA: Malondialdehyde; SOD: Superoxide dismutase; GPx: Glutathione peroxidase; IL-6: Interleukin-6; TNF-α: Tumor necrosis factor-α; GRP-78: Glucose-regulated protein-78; p-JNK: Phosphorylated c-Jun-N-terminal kinase; p-IRE1α: Phosphorylated Inositol-Requiring Transmembrane Kinase/Endoribonuclease 1α; JNK: C-jun-N-terminal kinase; Bax: BCL 2 associated X protein; Bcl-2: B-cell lymphoma 2; BTB: blood-testis barrier; ZO1: Zonula occludens-1; StAR: Steroidogenic acute regulatory protein; GSK-3α: Glycogen synthase kinase 3α; CatSper: Cation channels of sperm.
