# Supplementary material for: Cross-talk between ER stress and mitochondrial pathway mediated adriamycin-induced testicular toxicity and DA-9401 modulate adriamycin-induced apoptosis in Sprague–Dawley rats
Source: Cancer Cell Int. 2019 Apr 4;19:85. doi: 10.1186/s12935-019-0805-2 (PMC6450013; doi:10.1186/s12935-019-0805-2)
Supplement: Supplementary file 1 — Additional file 1: Fig. S1. Schematic diagram showing adriamycin (ADR)-induced testicular toxicity and its prevention by DA-9401 via suppression of oxidative stress, inflammation, endoplasmic reticulum (ER) stress, blood testis barrier (BTB), and apoptosis in the testis tissue. ER: Endoplasmic reticulum; ROS/RNS: Reactive oxygen species/reactive nitrogen species; MDA: Malondialdehyde; SOD: Superoxide dismutase; GPx: Glutathione peroxidase; IL-6: Interleukin-6; TNF-α: Tumor necrosis factor-α; GRP-78: Glucose-regulated protein-78; p-JNK: Phosphorylated c-Jun-N-terminal kinase; p-IRE1α: Phosphorylated Inositol-Requiring Transmembrane Kinase/Endoribonuclease 1α; JNK: C-jun-N-terminal kinase; Bax: BCL 2 associated X protein; Bcl-2: B-cell lymphoma 2; BTB: blood-testis barrier; ZO1: Zonula occludens-1; StAR: Steroidogenic acute regulatory protein; GSK-3α: Glycogen synthase kinase 3α; CatSper: Cation channels of sperm. [file 12935_2019_805_MOESM1_ESM.pptx]

## Slide 1
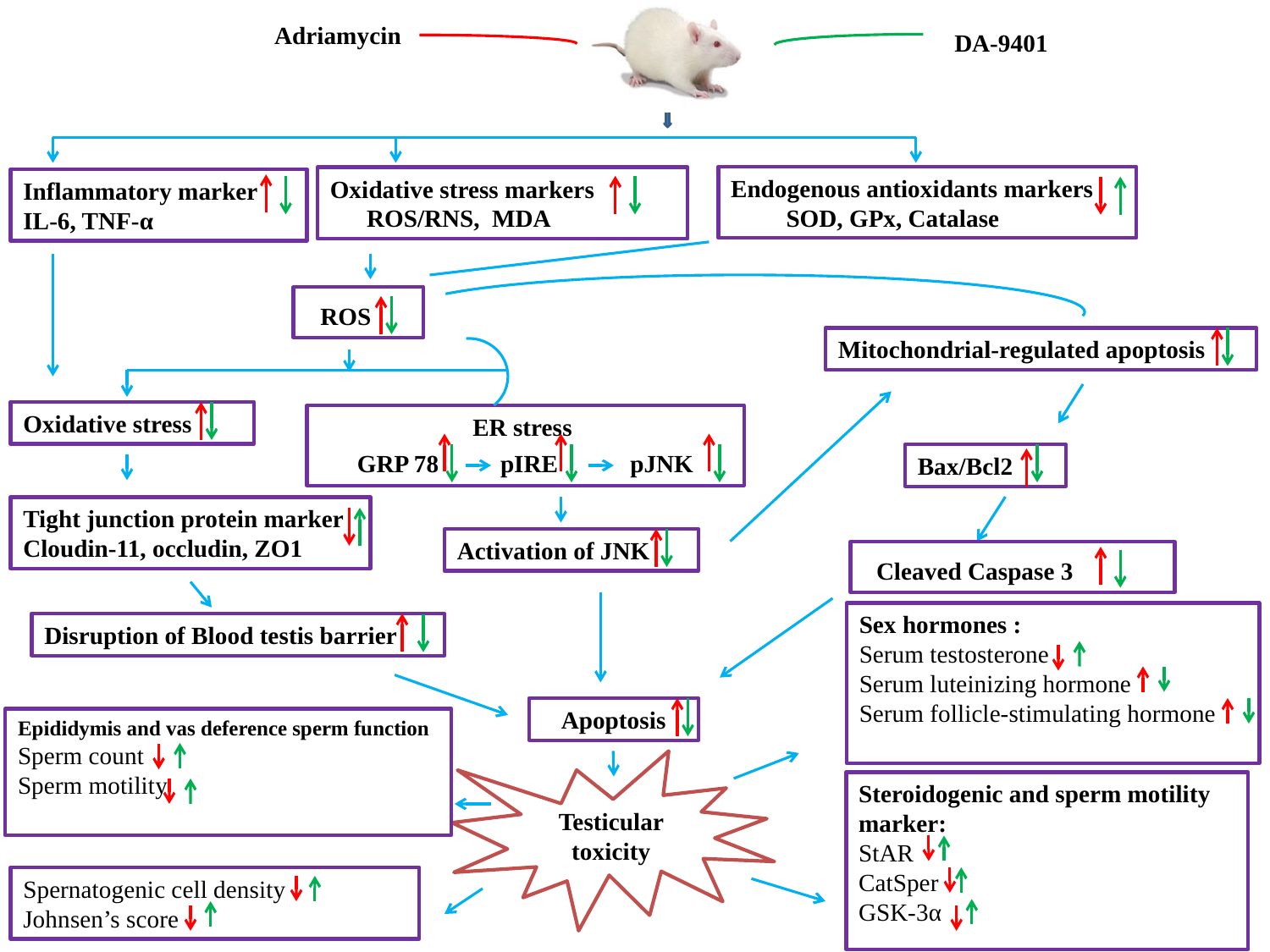

Adriamycin
DA-9401
Endogenous antioxidants markers
 SOD, GPx, Catalase
Oxidative stress markers
 ROS/RNS, MDA
Inflammatory marker
IL-6, TNF-α
 ROS
Mitochondrial-regulated apoptosis
Oxidative stress
ER stress
GRP 78 pIRE pJNK
Bax/Bcl2
Tight junction protein marker
Cloudin-11, occludin, ZO1
Activation of JNK
 Cleaved Caspase 3
Sex hormones :
Serum testosterone
Serum luteinizing hormone
Serum follicle-stimulating hormone
Disruption of Blood testis barrier
Apoptosis
Epididymis and vas deference sperm function
Sperm count
Sperm motility
Testicular toxicity
Steroidogenic and sperm motility marker:
StAR
CatSper
GSK-3α
Spernatogenic cell density
Johnsen’s score
